# Measurement of Oxidative Stress Index in 102 Patients with Peyronie’s Disease

**DOI:** 10.3390/metabo15080503

**Published:** 2025-07-29

**Authors:** Gianni Paulis, Andrea Paulis, Giovanni De Giorgio, Salvatore Quattrocchi

**Affiliations:** 1 Peyronie’s Care Center, Department of Urology and Andrology, Castelfidardo Clinical Analysis Center, 00185 Rome, Italy; 2 Bambino Gesù Children’s Hospital, IRCCS (Istituti di Ricovero e Cura a Carattere Scientifico), 00165 Rome, Italy; andrea.fx.94@gmail.com; 3 Section of Ultrasound Diagnostics, Department of Urology and Andrology, Castelfidardo Clinical Analysis Center, 00185 Rome, Italy; g.degiorgio@analisiclinichecastelfidardo.it; 4 Clinical Analysis Laboratory, Castelfidardo Clinical Analysis Center, 00185 Rome, Italy; salvatore.quattrocchi2@gmail.com

**Keywords:** Peyronie’s disease, oxidative stress, oxidative stress index, reactive oxygen species (ROS), total oxidant status, total antioxidant status, antioxidants

## Abstract

**Background**: *Peyronie’s disease* (PD) is a chronic inflammatory condition that affects the penile albuginea. Oxidative stress (OS) plays a crucial role in the development of the disease, prompting us to investigate OS levels at the site of the disease and in peripheral blood. This article presents our second study in which the OS was evaluated by calculating the OS index (OSI) in blood samples taken directly from the penile corpora cavernosa of patients with PD. Our innovative diagnostic method, which focuses on the analysis of oxidative stress (OS) in the corpora cavernosa of the penis, allows us to accurately identify the “chemical” signals (OS levels) of the pathology in the area where it is present. **Methods**: Our study included 102 PD patients from our Peyronie’s care center and 100 control cases. To conduct a comprehensive OS analysis, we measured both the total oxidant status (TOS) and total antioxidant status (TAS) and calculated the oxidative stress index (OSI) as OSI = TOS/TAS × 100. Blood samples were collected from the penis and a vein in the upper extremity, and OS was measured using d-ROMs and PATs (FRAS kit). **Results**: Pearson’s analyses revealed a significant statistical correlation between penile OSI values and PD plaque volumes (*p* = 0.003), while no correlation was found between systemic OSI values and plaque volumes (*p* = 0.356). Penile OSI values decreased significantly after PD plaque removal (*p* < 0.0001). A comparison of penile OSI values in PD patients (post plaque removal) and the control group showed no significant differences (*p* = 0.418). **Conclusions**: The lack of correlation between systemic OSI values and Peyronie’s plaque volume suggests that direct sampling from the site of the disease is preferable for OS studies. Conducting a penile OSI study could provide a precise oxidative marker dependent on plaque volume. In addition, the penile OSI study can biochemically monitor the therapeutic result, alongside penile ultrasound imaging.

## 1. Introduction

Peyronie’s disease (PD) is a chronic inflammatory condition that affects the penile tunica albuginea. The prevalence of PD ranges from 3.2% to 13% and is higher in Western countries [1,2,3,4]. This disease has a genetic predisposition with an autosomal dominant transmission [5,6]. PD results in the development of a fibrous and inelastic plaque that gradually grows and ultimately alters the shape of the penis, causing various types of deformations (curvature, shortening, depression, hollowing, and hourglass penis). In addition to the possible change in penile shape, PD can cause penile pain, erectile dysfunction (ED), and anxious–depressive states [7,8,9,10]. Although the exact pathogenesis of PD is not fully understood, the traumatic origin of the disease has been widely recognized [11,12,13,14,15]. The patient may also not remember the traumatic event because the penile trauma can also be minimal (microtrauma).

After a penile traumatic event, blood collection occurs. This blood is not reabsorbed, and this leads to the recruitment of inflammatory cells and fibrogenic factors, cytokines, and free radicals (oxidative stress), resulting in the local hyperproduction of collagen and the formation of a plaque [16,17,18,19,20,21,22,23,24]. PD occurs in two stages. The first stage corresponds to the initial phase (active phase), which lasts for about 12–18 months, during which plaque formation and growth occur [19]. The second stage of the disease corresponds to plaque stabilization. In this phase, the plaque, which is completely fibrotic and sometimes calcified (partially or completely), has stopped growing [25,26]. In this second stage, penile pain has disappeared permanently, and the deformity has stopped progressing and worsening.

During the first stage of the disease, conservative treatment is recommended. Conservative medical therapy consists of oral substances (vitamin E, potaba, colchicine, tamoxifen, pentoxifylline, and other antioxidants); phosphodiesterase 5 (PDE-5) inhibitors; anti-inflammatory drugs (NSAIDs); penile injections with antifibrogenic substances such as corticosteroids, verapamil, hyaluronic acid, interferon-α2b, pentoxifylline, and clostridium histolyticum collagenase (CCH); and physical therapies (iontophoresis, extracorporeal shock wave therapy, vacuum, and penile traction devices) [27,28,29,30,31,32,33,34,35,36,37,38,39,40,41]. In the second stage of PD, surgical intervention is recommended, including corporoplasty, plaque incision, and/or the implantation of a penile prosthesis [8,24,42,43,44,45,46,47,48,49].

Since oxidative stress (OS) is a fundamental component of the biochemical events of PD, we believe it is important that, in specific cases, the diagnosis of PD be integrated with the evaluation of OS in the cavernous bodies of the penis, directly at the site of the disease. In this manner, we take advantage of the presence of vascular tissue in the penis. In other pathological conditions, the search for oxidative stress markers is typically performed in peripheral blood samples [50,51,52,53,54].

Current diagnostic methods for PD mainly include the use of ultrasound and the manual palpation of the plaque. However, ultrasonography, if not performed with state-of-the-art equipment and suitable probes, may not provide us with an accurate picture of the disease present in the penis. Studying oxidative stress in the penis can instead allow us to precisely evaluate the local chemical situation, which is the direct expression of the diseased area and its extent.

Our innovative diagnostic method, which focuses on analyzing the OS in the cavernous bodies of the penis, enables us to precisely identify the “chemical” signs of disease regression in the area where it was previously detected.

## 2. Materials and Methods

### 2.1. Study Population

This study involved 102 patients diagnosed with PD who were examined at our Peyronie’s Care Center between June 2017 and March 2025. A control group of 100 healthy individuals without PD or other organic pathologies was also included in this study. It was conducted in accordance with the Declaration of Helsinki (Fortaleza, 2013), and all participants provided informed consent. Sensitive data were anonymized in compliance with privacy regulations, including LEGISLATIVE DECREE 10 August 2018, no. 101, adapted to the GDPR. We analyzed existing data from all patients, including those in the control group, by reviewing medical records from our andrology clinic for this study on OS.

We specifically focused on the analysis of data related to this study of OS in PD patients before starting treatment with antioxidants and also after treatment. However, these treatments are not the subject of this article.

### 2.2. Purpose of This Study

Oxidative stress (OS) can be assessed by measuring the blood levels of reactive oxygen metabolites (ROMs) (total oxidant status/TOS) and total antioxidant status (TAS).

We used the d-ROM test and the plasma antioxidant test (PAT) to determine these values [55,56].

For an accurate assessment of OS, it is essential to consider the individual measurements of d-ROMs and PAT as well as their ratio, known as the OS index (OSI, arbitrary unit) and calculated as OSI = d-ROMs/PAT × 100 [57,58,59,60].

In most cases, research on OS in different diseases is carried out using blood samples obtained from a peripheral vein. However, this method only provides an analysis of the “overall” oxidative state of the patient [57,58,59,61,62,63,64].

The main aim of this research was to evaluate the levels of free radicals (d-ROM) and antioxidant capacity (PAT) in the peripheral blood and specifically in the penile corpora cavernosa of PD patients to directly assess OS in the affected area.

Another important aim of this study is to determine if treatment with antioxidants leads to a reduction in OS, as has already been reported in other pathologies [62].

This study involved 102 PD patients who had already shown ultrasound evidence of the disappearance of the PD plaque. To determine the current status of the OS, we computed the OS index using a specific formula (d-ROMs/PAT × 100).

The additional objectives of this research include the following:

• The assessment of the OS index in PD patients and in the subjects of the control group;
• The investigation of a potential relationship between the penile OSI values and the penile plaque volumes;
• The investigation of a potential relationship between systemic OSI values (obtained from blood samples from a peripheral vein) and plaque volumes;
• The investigation of a potential relationship between systemic OSI values and penile OSI values;
• The identification of normal penile OSI values indicative of disease area regression (PD plaque);
• The investigation of a potential correlation between penile OSI values and chronic pathological conditions;
• The investigation of a potential correlation between systemic OSI values and chronic pathological conditions in progress;
• The investigation of a potential correlation between penile OSI values and an anxious–depressive state;
• The investigation of a potential correlation between systemic OSI values and an anxious–depressive state.

#### 2.2.1. Inclusion and Exclusion Criteria

Inclusion criteria: All patients with PD included in this study were between 21 and 70 years of age. They had complete data from a comprehensive examination of their medical history, including information on all existing medical conditions. Their diagnosis of PD was obtained through the palpation of the penis, the acquisition of photographs of penile deformity (according to Kelâmi), the goniometric measurement of penile angulation, and a color Doppler ultrasound of the penis, with measurements of the plaque in three dimensions (in mm), and by calculating the volume (mm^3^) using the ellipsoidal formula (volume = 0.524 × length × width × thickness). The patients also completed the International Index of Erectile Function (IIEF) questionnaire to assess erectile function, the visual analog scale (VAS) questionnaire to measure pain, the Generalized Anxiety Disorder-7 (GAD-7) questionnaire to assess levels of anxiety, and the Patient Health Questionnaire-9 (PHQ-9) to assess the levels of depression [65,66,67,68,69,70,71].

All PD patients were free of the following conditions (ongoing or recent (within the last three months)): infection (bacterial, viral, or other), acute or chronic inflammatory diseases, thyroid hyperfunction, hypertensive crisis, obesity, ischemic and/or infarct episode, renal failure, diabetes mellitus, neoplastic disease, liver dysfunction, periodontal dental treatment, surgery, and any other conditions that disrupt the normal redox balance.

The subjects of the control group were required to complete the following questionnaires: IIEF, PHQ-9, GAD-7, and VAS.

The 102 patients with PD and the control group members were required to undergo an oxidative stress study (d-ROMs and PAT) with the subsequent calculation of the OSI. Blood samples were taken directly from the penile corpora cavernosa using a 25 G needle and from the peripheral blood (standard sampling from a vein in the upper extremity).

The patients were required to fast in the evening prior to the oxidative stress study.

Each PD patient was required to undergo an OS study before and after receiving antioxidant treatment, with follow-up after each treatment cycle.

More than one check-up after treatment was required, with at least one check-up occurring at least 6 months after the treatment period. For each follow-up visit, the patients were required to temporarily stop taking antioxidants for at least 10 days before undergoing blood tests.

Exclusion criteria: All PD patients with the following conditions (ongoing or recent (within the last three months)) were excluded: infection (bacterial, viral, or other), acute or chronic inflammatory diseases, thyroid hyperfunction, hypertensive crisis, obesity, ischemic and/or infarct episode, renal failure, diabetes mellitus, neoplastic disease, liver dysfunction, periodontal dental treatment, surgery, and any other conditions that disrupt the normal redox balance.

A total of 102 patients who had undergone an OS study and met the inclusion criteria were selected from the overall group of PD patients treated at our care center and enrolled in this study. All 102 patients successfully completed the treatment, resulting in the disappearance of the disease.

All PD patients underwent OS examination before and after treatment, with follow-up after each treatment cycle.

The PD patients received the following treatment: oral L-carnitine, 1000 mg; bilberry, 180 mg; propolis, 700 mg; ginkgo biloba, 240 mg; silymarin, 400 mg; coenzyme Q-10, 100 mg; vitamin C, 50 mg; vitamin E, 48 mg; and superoxide dismutase, 11,000 IU/g 10 mg daily. They also used topical diclofenac gel 4% twice daily and received peri-plaque penile injections (only for plaques with volume ≥ 100 mm^3^) with pentoxifylline 100 mg (30 G needle) monthly for 12 months, followed by 1 injection every 2 months for 12 months (18 total injections).

Due to the difficulty of finding cases for the control group, we included 100 subjects without PD and chronic inflammatory and/or degenerative diseases whom we visited at our andrological clinic, where we diagnosed primary non-organic premature ejaculation (PE).

#### 2.2.2. Data Collection

We collected demographic information on PD patients, as well as ultrasound details (plaque location, size, and volume), the type and degree of penile curvature, and the degree of erectile dysfunction and penile pain. We also documented the demographic and clinical data of the 100 control subjects.

#### 2.2.3. Sample Collection

Blood samples were taken from both the penile corpora cavernosa and a vein in the upper extremity. A syringe and 25 G needle were used to collect blood from the penile corpora cavernosa, avoiding puncturing the PD plaque. This procedure is not painful, so local anesthesia was not required. Approximately 0.5 mL (500 µL) of blood was collected from the penile corpora cavernosa. Blood was also collected from the peripheral vein in the upper extremity using a standard method, with approximately 1–2 mL of blood aspirated to ensure at least 0.5 mL (500 µL) for the examination. The blood samples were immediately transferred to a heparinized tube (not an EDTA tube).

#### 2.2.4. Plasma Collection

The samples were centrifuged at 1600 rpm for 90 s to separate the plasma from the rest of the sample. Following this, 10 µL of plasma samples was reacted with the appropriate reagents for the d-ROM test and PAT. The cuvettes containing the samples were then inserted into the photometric analytical device for analysis.

In this study, we utilized the FRAS 5 machine along with its associated kits [60].

#### 2.2.5. d-ROMs and PAT Measurements

The d-ROMs Fast test was utilized to measure the concentration of peroxides, while the plasma antioxidant test (PAT) was used to measure antioxidant capacity; both tests are components of the FRAS kit (Parma, IT) [60].

The d-ROM test used Carratelli units (Carr. U.) as the unit of measurement, while the PAT used Cornelli units (Cor. U.) [55,56]. Normal values for d-ROMs are between 250 and 300 Carr. U., and for PAT, normal values range from 2200 to 2800 Cor. U., with values below 1800 Cor. U. considered deficient [55,56].

The OSI was calculated using the following formula: OSI (arbitrary unit) = d-ROMs/PAT × 100 [55,56,57,58,59].

#### 2.2.6. Statistical Analysis

We utilized the MedCalc statistical software (version 16.4.3, 2016) to conduct the t-test and chi-square test.

CalculatorSoup^®^ software (version of 8 March 2023) was employed to calculate the standard deviation, mean, median, and interquartile range (IQR).

SPSS Statistics software version 22.0 (2013) was used to determine the Pearson correlation coefficient, conduct the Mann–Whitney U test (Wilcoxon rank sum), and perform the Shapiro–Wilk test to assess the normality of the values.

To assess the diagnostic accuracy of penile OSI and generate a ROC curve, we utilized Eng J’s ROC analysis tool available on the Johns Hopkins University website (version as of 17 February 2022), accessed via the link “http://www.rad.jhmi.edu/jeng/javarad/roc/JROCFITi.html” on 2 June 2025.

To determine the optimal cutoff point for measuring the sensitivity and specificity of the test in relation to the ROC curve (Youden index), we utilized the Youden index calculator (MDApp, version dated 29 June 2020, Manchester, UK).

For the statistical analysis of logistic regression, we used AgriMetSoft’s software (version 2023) and Excel (MS Office, Redmond, WA, USA, version 2024).

A significance level of 5% for the alpha error (*p*-value < 0.05) was applied to determine statistical significance.

## 3. Results

Table 1 shows the demographic and clinical characteristics of the PD patients and control group subjects.

There were no significant differences in the demographics between the group of PD patients and the control group subjects (see Table 2).

However, there are statistically significant differences for some clinical conditions between the groups.

The values of the d-ROMs, PAT, and the relative OS index (both systemic and penile) for the PD patients and control group subjects can be found in Tables S1 and S2 in the Supplementary Materials.

The Pearson’s correlation coefficient analysis did not show a significant correlation between the systemic OSI values and the penile OSI values of the PD patients (Pearson’s correlation coefficient *p*-value = 0.954). Additionally, a statistically significant difference was found between the systemic OSI values and the penile OSI values using the t-test, with a *p*-value < 0.0001.

**Figure 1:**
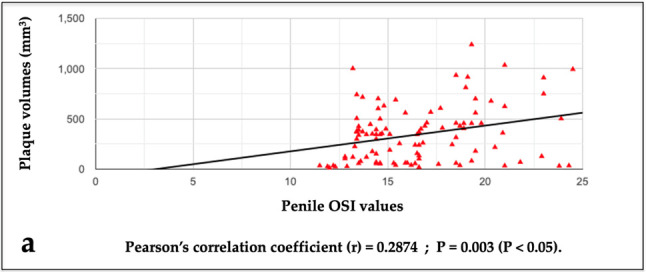

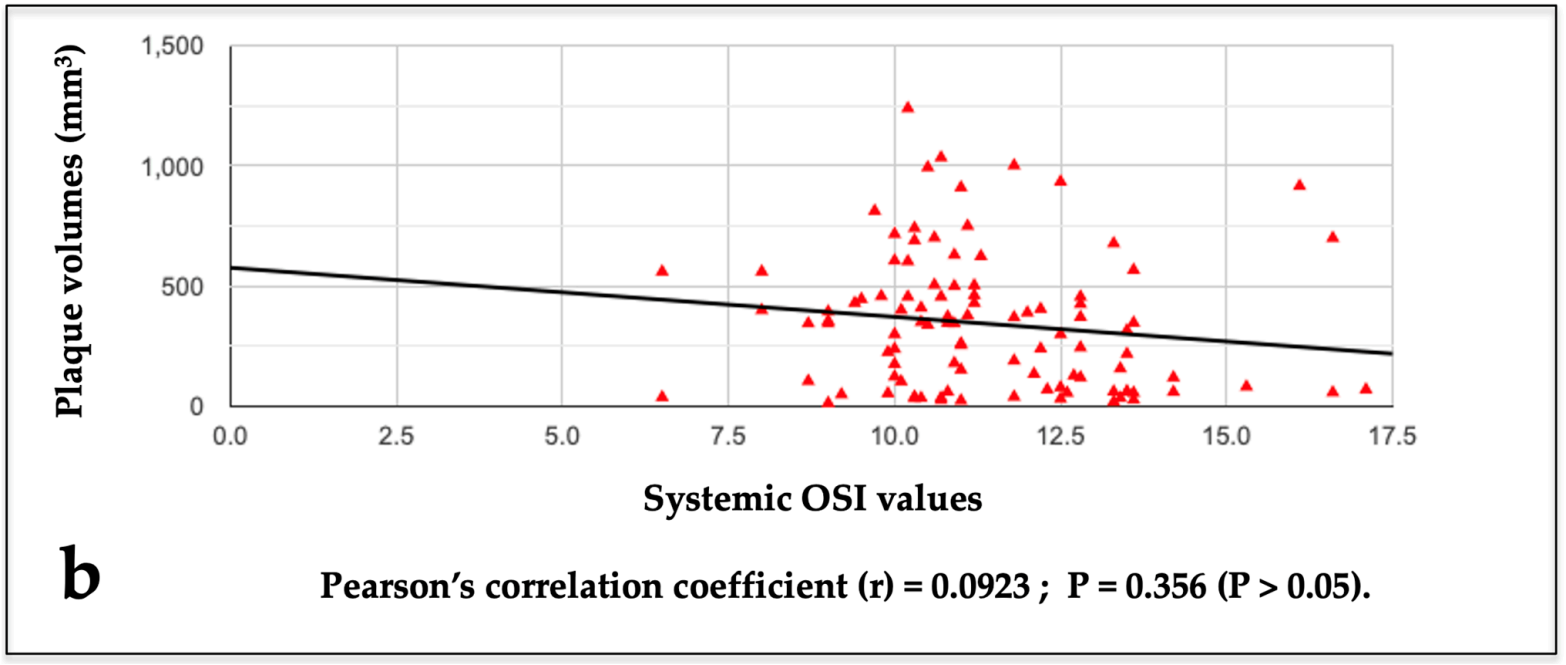
Graph highlighting the relationship between plaque volumes and penile OS index values (**a**) and between plaque volumes and systemic OS index values (**b**).

The Pearson’s correlation coefficient (PCC) analysis showed a high significant statistical correlation between the penile OSI values and the volumes of the disease area (PD plaque): PCC = r 0.2874, r^2^ 0.08261, *p*-value = 0.003 (see Figure 1a).

This analysis did not show a significant statistical correlation between the systemic OSI values and the volumes of the disease area (PD plaque): PCC = r 0.0923, r^2^ 0.0085, *p* = 0.356 (see Figure 1b).

No correlation has been established between OSI values (both systemic and penile) and associated pathological conditions, including cigarette smoking and anxious–depressive state (see Table 2).

There were statistically significant differences in the penile OSI values between the PD patients (before treatment) and the control group subjects (*p*-value < 0.0001) (see Table 3).

We found that the penile OSI values decreased significantly after treatment and specifically after the elimination of the PD plaque (t-test, *p*-value < 0.0001).

**Figure 2:**
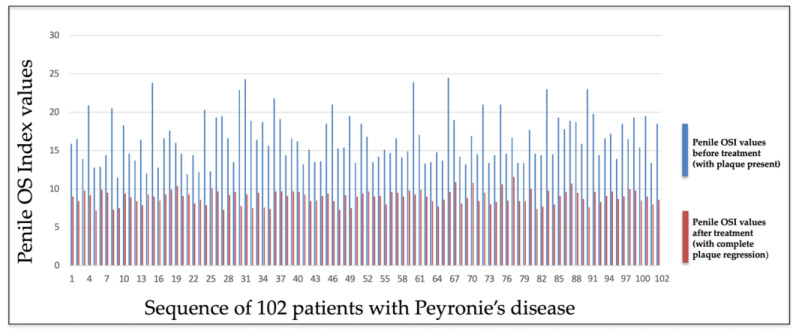
OS index values of penile corpora cavernosa before and after the treatment and disappearance of the PD plaque.

Figure 2 illustrates the decrease in the penile OSI values after the treatment and the disappearance of Peyronie’s plaque.

In our study, we found that in PD patients (after treatment and plaque disappearance), the mean penile OSI was 8.95 ± 0.90. In the control group subjects, a mean value of the penile OSI of 9.1 ± 1.65 was obtained.

No statistically significant differences were found when comparing the penile OSI values of PD patients who achieved plaque disappearance after treatment with those of the control group subjects (t-test, *p*-value = 0.418).

**Figure 3:**
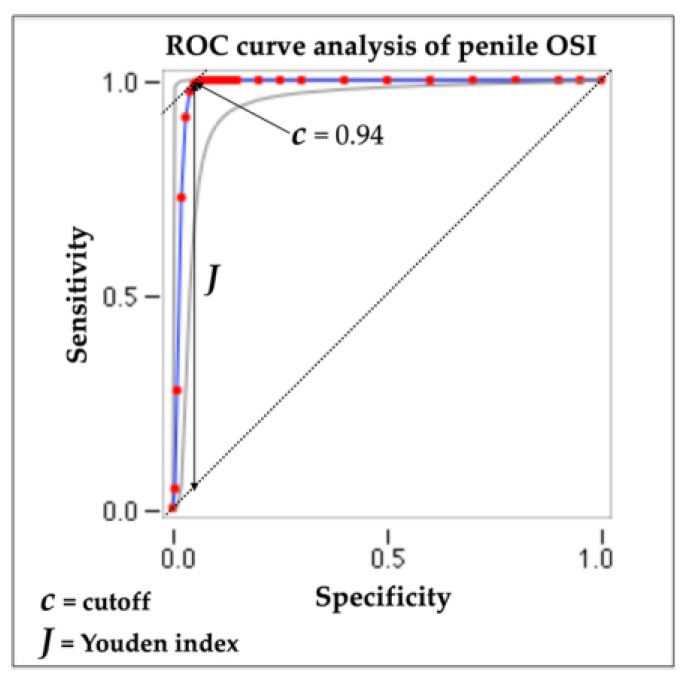
ROC curve analysis of penile OSI and determination of optimal cutoff point (Youden index).

The ROC curve analysis revealed an area under the curve of 0.984, an accuracy of 97.0%, a sensitivity of 99.0%, and a specificity of 95%. Additionally, the optimal cutoff value for penile OSI was determined to be 0.94 based on the calculation of the Youden index (see Figure 3).

Based on all the statistical results, we identified that the normal values of penile OSI should be ≤11.53; doubtful values, from 11.54 to 11.9; and pathological values, ≥12.0.

Table 4 presents the findings on the prevalence of depression and anxiety among the 102 PD patients.

## 4. Discussion

The OS index (OSI), first introduced in 2003 by Erel et al. [57], increases in correlation with any oxidative imbalance [57,58,59].

This article presents our second study in which OS was evaluated by calculating the OS index (OSI) in blood samples taken directly from the penile corpora cavernosa of patients with PD [54]. The search for oxidative stress markers directly at the site affected by the disease has been described only in limited cases, such as with periodontitis and ocular rosacea [50,51]. In this study, the patient sample was significantly larger, as was the control group. In our previous study, the control group consisted of subjects with purely psychological ED who had undergone various diagnostic tests that ruled out an organic or vascular origin of ED [54]. However, since it has been reported in the literature that patients with psychogenic ED have an overproduction of cortisol and proinflammatory cytokines, we decided to use another control group consisting exclusively of patients without chronic inflammatory and/or degenerative diseases whom we visited at our andrological clinic where we diagnosed primary non-organic premature ejaculation (PE) [72,73]. Until recently, the biological antioxidant potential (BAP) test was commonly used to measure antioxidant capacity. This test is based on plasma’s ability to reduce ferric ions to ferrous ions. This method (BAP test) is subject to evaluation bias because the colorimetric determination of ferric ions can be affected by the presence of phosphate ions. In the plasma sample, some of the ferric ions that are complexed with thiocyanate ions may interact with the phosphate ions, leading to the decolorization of the solution. This decolorization is not dependent on the actual concentration of antioxidants but rather on the concentration of phosphate ions. The outcome of this chemical reaction of the test leads to the significant overestimation of the antioxidant capacity.

In this study and our previous study, we employed the latest and updated PAT method, which also relies on the colorimetric evaluation of ferric ions and incorporates zirconium salt at appropriate levels [55]. Zirconium is a transition metal that forms a stronger bond with phosphates, effectively removing interference from plasma phosphates. The findings of our present research support and enhance the understanding that OS plays a significant role in the pathogenesis and development of PD [16,17,18,19,20,21,22,23,24,74]. Penile OSI may be viewed as a biochemical parameter for assessing the severity and extent of PD, as our two studies revealed a strong correlation between penile OSI values and PD plaque volume.

It is important to understand that although various inflammatory cells and proinflammatory cytokines are active during chronic inflammation, the primary factor determining this state is OS.

OS generates large amounts of reactive oxygen species (ROS), initiating and sustaining the biochemical process of this inflammation, ultimately promoting disease progression and the gradual growth of PD plaque.

The correlation between local OSI levels and plaque volume, as detected in our study, demonstrates that oxidative stress is essential for the initiation, maintenance, and progression of inflammation.

Further evidence of this is the progressive reduction in the inflammatory area (plaque) that can be achieved after treatment with antioxidants.

By identifying a strong correlation between the size of Peyronie’s plaques and penile OSI values, we can use ultrasound to measure PD plaque volume and the penile OSI to confirm the actual presence and local extent of the disease. In addition, the study of penile OSI can help us biochemically monitor the therapeutic results, alongside penile ultrasound imaging.

Additionally, if a certain ultrasound diagnosis is not available, the examination of OS in the penile corpora cavernosa could serve as a more certain and conclusive diagnostic test.

The absence of a relationship between the size of Peyronie’s plaque and the systemic OSI suggests that for a study of OS to be truly useful, samples must be taken directly from the site of the disease, as performed in previous studies on other disease conditions [50,51,75].

The lack of statistically significant differences in penile OSI values between PD patients with plaque removal and control group subjects suggests that penile OSI testing can be an effective method for studying oxidative markers in this disease. This was not the aim of our study; note that our results demonstrate the elimination of plaque after antioxidant treatment, as already described in the literature [76,77,78,79,80].

Although this study did not show a correlation between penile OSI values and anxiety/depressive status, we feel it is fair to point out that, unfortunately, we found a significant number of PD patients suffering from anxiety (78.4%) and depression (56.8%). These findings confirm that they are common symptoms of Peyronie’s disease, along with penile pain, curvature or simple penile deformation, and erectile dysfunction [10,81,82,83]. Other studies using the Peyronie’s Disease Questionnaire (PDQ) have also reported high percentages of anxiety and depression [84].

Although our results are interesting, an objective limitation of our study is that it is not possible to compare our results with those of other similar studies by other authors, as the literature in this field of research contains only our recent previous study.

Further research is needed to investigate oxidative stress in the penile corpora cavernosa of patients with PD. To enhance this clinical investigation, animal models could be utilized in experimental studies. For instance, conducting experimental studies on rats to induce penile fibrosis by injecting fibrogenic agents (TGF-β, fibrin, or others) into the penile tunica albuginea, as demonstrated in previous studies, would be essential [85,86,87]. In these possible experimental studies, following the development of fibrotic regions in the penis, oxidative stress levels could be assessed in both the corpora cavernosa and peripheral blood of rats. Subsequently, antioxidants or antifibrotics (anthocyanin, platelet-rich plasma, or others) could be administered into the penile corpora cavernosa of rats to mitigate fibrosis [87]. Once this outcome is achieved, the OSI can be measured in the same manner as before the treatment.

## 5. Conclusions

The lack of a relationship between the PD plaque volume and systemic OSI and, conversely, the presence of a significant correlation between the PD plaque volume and penile OSI value suggests that an assessment of oxidative stress should be conducted directly at the site of the disease. This has been demonstrated by other researchers in various specialized medical fields. Combining penile OSI analysis with ultrasound examination to assess plaque volume could provide a valuable oxidative marker indicating the actual biochemical presence of the disease. While our findings are very interesting, further research is necessary to explore oxidative stress (including calculating the OSI) in the penile corpora cavernosa of PD patients.

## Figures and Tables

**Table 1:** Demographic and clinical characteristics of PD patients and control group subjects.

	Group of PD Patients (no. 102) Mean Age 49.01 Years (SD ± 11.50)		Control Group (no. 100) Mean Age 49.12 Years (SD ± 11.23)		Statistical Analysis PD Group Versus Control Group (*t*-Test) *p*-Value = 0.950
**Demographic** **characteristics**	No. of patients (out of 102) (%)		No. of patients (out of 100) (%)		***p*****-value** (chi-square test)
**Race**					
Caucasian	102 (100%)		100 (100%)		1.000
**Age Range**					
From 20 to 40 years	25 (24.50%)		26 (26.0%)		0.807
From 41 to 70 years	77 (75.49%)		74 (74.0%)		0.807
**Clinical condition** **associated with PD patients**	No. of patients (out of 102) (%)		No. of patients (out of 100) (%)		**Statistical analysis** **Group of PD patients** **versus** **Control group** ***p*****-value** (chi-square test)
Penile curvature	94 (92.1%)	Average penile curvature Angle (degrees) = 35.07°	0	Average penile curvature Angle (degrees) = 0°	<0.0001
Penile pain	54 (52.9%)	Mean VAS score = 4.4	0	Mean VAS score = 0	<0.0001
Erectile dysfunction	40 (39.2%)	Mean IIEF score = 21.1	0	Mean IIEF score = 26.5	<0.0001
Significant anxiety	80 (78.4%)	Mean GAD-7 score = 16.4	18 (18%)	Mean GAD-7 score = 7.4	<0.0001
Significant depression	58 (56.8%)	Mean PHQ-9 score = 15.2	6 (6.0%)	Mean PHQ-9 score = 2.6	<0.0001
Cigarette smoking	34 (33.3%)	Mean no. of cigarettes per day = 9.2	32 (32.0%)	Mean no. of cigarettes per day = 8.9	0.891

**NOTE**: PD = Peyronie’s disease; SD = standard deviation; *p*-value considered significant when <0.05. IIEF = International Index of Erectile Function. IIEF score range: severe erectile dysfunction (ED) (0–10), moderate ED (11–16), mild to moderate ED (17–21), mild ED (22–25), and no erectile dysfunction (26–30). VAS = visual analog scale. VAS score range: 1–5 for mild to moderate pain, 6–7 for severe pain, and 8–10 for very severe pain. GAD-7 = Generalized Anxiety Disorder-7 questionnaire. GAD-7 score range: minimal or mild anxiety, 1–9; moderate anxiety, 10–14; and severe anxiety, 15–21. Significant anxiety is present when the GAD-7 score is >9. PHQ-9 = Patient Health Questionnaire-9. PHQ-9 score range: minimal or mild depression, 1–9; moderate depression, 10–14; moderately severe depression, 15–19; and severe depression, 20–27. Significant depression is present when the PHQ-9 score is >9.

**Table 2:** Associated clinical conditions in 102 patients with Peyronie’s disease and the study of their possible statistical correlation with the “OS index values of the penile corpora cavernosa” or “systemic OS index values”.

Clinical Condition Associated with PD Patients	No. of Patients (out of 102)	Correlation with Penile OS Index Values (YES or NO)		Correlation with Systemic OS Index Values (YES or NO)	
Penile pain	54 (52.9%)	Pearson’s correlation coefficient (*p* = 0.562)	NO	Pearson’s correlation coefficient (*p* = 0.179)	NO
Penile curvature	94 (92.1%)	Pearson’s correlation coefficient (*p* = 0.245)	NO	Pearson’s correlation coefficient (*p* = 0.621)	NO
Erectile dysfunction	40 (39.2%)	Pearson’s correlation coefficient (*p* = 0.384)	NO	Pearson’s correlation coefficient (*p* = 0.327)	NO
Significant anxiety	80 (78.4%)	Pearson’s correlation coefficient (*p* = 0.795)	NO	Pearson’s correlation coefficient (*p* = 0.926)	NO
Significant depression	58 (56.8%)	Pearson’s correlation coefficient (*p* = 0.575)	NO	Pearson’s correlation coefficient (*p* = 0.231)	NO
Cigarette smoking	34 (33.3%)	Pearson’s correlation coefficient (*p* = 0.927)	NO	Pearson’s correlation coefficient (*p* = 0.895)	NO

**Table 3:** Blood levels of oxidative stress markers in the penile corpora cavernosa of patients with Peyronie’s disease and control subjects. Relative oxidative stress indices and statistical comparison.

	PD Patients (no. = 102) Mean (SD±)	Control Group (no. = 100) Mean (SD±)	Statistical Analysis *p*-Value (*t*-Test)
**d-ROM values** (Carr. U.)	487.5 (±91.4)	327.67 (±75.85)	<0.0001
**PAT values** (Cor. U.)	3011.77 (±558.06)	3487.76 (±595.61)	<0.0001
**OS Index (OSI)**	16.49 (±3.15)	9.1 (±1.65)	<0.0001

**NOTE:** PD = Peyronie’s disease; SD = standard deviation; Carr. U = Carratelli units; Cor. U = Cornelli units; d-ROMs = hydrogen peroxides; PAT = plasma antioxidant test; OS index (OSI) = oxidative stress index.

**Table 4:** Prevalence of depression and anxiety among 102 patients with PD.

PHQ-9 Score Range		No. Total Cases (%)
No depression	0	4 (3.9)
Minimal depression	1–4	6 (5.8)
Mild depression	5–9	34 (33.3)
Moderate depression	10–14	32 (31.3)
Moderately severe depression	15–19	22 (21.5)
Severe depression	20–27	4 (3.9)
**Significant** **depression**	**10–27**	**58 (56.8)**
**TOTAL**		**102 (100)**
**GAD-7 Score Range**		**No. Total Cases (%)**
No anxiety	0	0
Minimal or mild anxiety	1–9	22 (21.5)
Moderate anxiety	10–14	40 (39.2)
Severe anxiety	15–21	40 (39.2)
**Significant anxiety**	**10–21**	**80 (78.4)**
**TOTAL**		**102 (100)**

**NOTE**: PD = Peyronie’s disease. GAD-7 = Generalized Anxiety Disorder-7 questionnaire. GAD-7 score range: minimal or mild anxiety, 1–9; moderate anxiety, 10–14; and severe anxiety, 15–21. Significant anxiety is present when the GAD-7 score is >9. PHQ-9 = Patient Health Questionnaire-9. PHQ-9 score range: minimal or mild depression, 1–9; moderate depression, 10–14; moderately severe depression, 15–19; and severe depression, 20–27. Significant depression is present when the PHQ-9 score is >9.

## Data Availability

The original contributions presented in the study are included in the article/supplementary material. Further inquiries can be directed to the corresponding author.

## Supplementary Materials

The following supporting information can be downloaded at: https://www.mdpi.com/article/10.3390/metabo15080503/s1, Table S1: Values of d-ROM, PAT, and relative OSI (“systemic” and “in penile corpora cavernosa”) of the 102 PD patients. Table S2: Values of d-ROM, PAT, and relative OSI (“systemic” and “in penile corpora cavernosa”) of the 100 control cases (control group).
