# Supplementary material for: Measurement of Oxidative Stress Index in 102 Patients with Peyronie’s Disease
Source: Metabolites. 2025 Jul 29;15(8):503. doi: 10.3390/metabo15080503 (PMC12388598; doi:10.3390/metabo15080503)
Supplement: Supplementary file 1 [file metabolites-15-00503-s001.zip › metabolites-3741741-supplementary.pdf]

Table S1. Values of d-ROMs, PAT and relative OSI index ("systemic" and "in penile corpora cavernosa") of the 102 PD patients

	**d-ROMs values** Carratelli units (Carr. U.) systemic plasma sample	**PAT values** Cornelli units (Cor. U.) systemic plasma sample	**OSI index** systemic plasma sample	**d-ROMs values** Carratelli units (Carr. U.) penile plasma sample	**PAT values** Cornelli units (Cor. U.) penile plasma sample	**OSI index** penile plasma sample
1.	360	4242	**8.7**	414	3125	**13.2**
2.	354	3778	**9.4**	570	2949	**19.3**
3.	352	3283	**10.7**	390	2358	**16.5**
4.	498	4619	**10.7**	569	2910	**19.5**
5.	428	3841	**11.1**	395	2825	**13.9**
6.	344	3410	**10**	447	2680	**16.6**
7.	441	4057	**10.8**	616	2934	**20.9**
8.	470	3545	**13.6**	352	2590	**13.5**
9.	339	3055	**11**	543	2368	**22.9**
10.	282	2532	**11.1**	702	2885	**24.3**
11.	502	3114	**16.1**	410	3183	**12.8**
12.	539	3152	**17.1**	401	3102	**12.9**
13.	424	3176	**13.3**	513	3557	**14.4**
14.	354	2756	**12.8**	605	2941	**20.5**
15.	390	4288	**9**	412	3562	**11.5**
16.	390	3080	**12.6**	535	2825	**18.9**
17.	321	2631	**12.2**	439	2389	**18.3**
18.	291	3157	**9.2**	438	2665	**16.4**
19.	358	2576	**13.8**	508	2716	**18.7**
20.	352	2842	**12.3**	464	2964	**15.6**
21.	580	4080	**14.2**	560	2564	**21.8**
22.	342	2885	**11.8**	416	2840	**14.6**
23.	392	3245	**12**	503	2631	**19.1**
24.	414	2643	**16.5**	403	2798	**14.4**
25.	246	1826	**13.4**	464	2787	**16.6**
26.	205	3123	**6.5**	479	2941	**16.2**
27.	280	2821	**9.9**	382	2885	**13.2**
28.	418	3042	**13.7**	415	2737	**15.1**
29.	438	2830	**15.4**	436	3216	**13.5**
30.	485	3162	**15.5**	399	2892	**13.7**
31.	245	3052	**8**	400	2430	**16.4**
32.	324	2641	**12.2**	448	3719	**12**
33.	314	3218	**9.7**	725	3045	**23.8**
34.	341	3353	**10.1**	506	3936	**12.8**
35.	422	3157	**13.3**	399	2928	**13.6**
36.	326	2641	**12.1**	572	3440	**16.6**
37.	339	3096	**10.9**	498	2823	**17.6**
38.	300	2861	**10.4**	551	3442	**16**
39.	603	3937	**15.3**	521	3564	**14.6**
40.	387	3526	**10.9**	555	2991	**18.5**
41.	498	2990	**16.6**	534	2537	**21**
42.	379	2842	**13.3**	612	3985	**15.3**
43.	338	2685	**12.5**	410	2652	**15.4**
44.	330	2034	**10.8**	425	3564	**11.9**
45.	319	3080	**10.3**	409	2840	**14.4**
46.	359	2806	**12.7**	563	4608	**12.2**
47.	435	2464	**17.6**	525	2685	**19.5**
48.	468	4199	**11.1**	538	2641	**20.3**
49.	538	3576	**15**	384	3111	**12.3**
50.	460	4243	**10.8**	414	3080	**13.4**
51.	432	3776	**11.4**	570	3065	**18.5**
52.	512	3298	**15.5**	390	2311	**16.8**
53.	523	4655	**11.2**	569	4200	**13.5**
54.	435	3878	**11.2**	395	2766	**14.2**
55.	444	3555	**12.4**	447	2960	**15.1**
56.	540	4123	**13**	616	4190	**14.7**
57.	470	3545	**13.2**	352	2111	**16.6**
58.	339	3055	**11**	543	3834	**14.1**
59.	324	2530	**12.8**	702	4689	**14.9**
60.	506	3111	**16.2**	410	1711	**23.9**
61.	530	3150	**16.8**	401	2333	**17**
62.	421	3089	**13.6**	513	3849	**13.3**
63.	359	2777	**12.9**	605	4481	**13.5**

64.	512	4321	**11.8**	412	2780	**14.8**
65.	432	3114	**13.8**	535	3898	**13.7**
66.	399	2621	**15.2**	439	2389	**24.5**
67.	433	3113	**13.9**	399	2100	**19**
68.	456	2554	**17.8**	429	3001	**14.2**
69.	351	2855	**12.2**	384	2899	**13.2**
70.	456	4065	**11.2**	508	2989	**16.9**
71.	349	2877	**12.1**	414	2841	**14.5**
72.	399	3045	**13.1**	539	2559	**21**
73.	402	2622	**15.3**	402	2997	**13.4**
74.	240	1833	**13**	430	2980	**14.4**
75.	421	3165	**13.3**	500	2378	**21**
76.	281	2811	**9.9**	449	3069	**14.6**
77.	411	3021	**13.6**	409	2437	**16.7**
78.	456	2899	**15.7**	407	3036	**13.4**
79.	485	3162	**15.3**	390	2891	**13.4**
80.	321	3046	**10.5**	410	2314	**17.7**
81.	312	2655	**11.7**	448	3059	**14.6**
82.	432	3200	**13.5**	564	3894	**14.4**
83.	366	3378	**10.8**	755	3272	**23**
84.	435	3199	**13.5**	377	2600	**14.5**
85.	318	2676	**11.8**	640	3307	**19.3**
86.	333	3099	**10.7**	499	2803	**17.8**
87.	342	2802	**12.2**	551	3442	**18.9**
88.	569	3999	**14.2**	669	3563	**18.7**
89.	398	3511	**11.3**	514	3230	**15.9**
90.	434	2967	**14.6**	576	2499	**23**
91.	334	2866	**11.6**	732	3685	**19.8**
92.	312	2614	**11.9**	400	2777	**14.4**
93.	265	2037	**13**	509	3064	**16.6**
94.	316	3047	**10.3**	477	2769	**17.2**
95.	367	2811	**13**	599	4308	**13.9**
96.	398	2566	**15.5**	496	2670	**18.5**
97.	368	3199	**11.5**	481	2899	**16.5**
98.	465	3123	**14.8**	535	2767	**19.3**
99.	444	3541	**12.5**	450	2922	**15.4**
100.	419	3200	**13**	509	2600	**19.5**
101.	343	2554	**13.4**	404	3003	**13.4**
102.	335	2776	**12**	402	2170	**18.5**

NOTE : d-ROMs = derived from the Reactive Oxygen Metabolites; PAT = Plasma Antioxidant Test; OSI = Oxidative Stress Index.

Table S2. Values of d-ROMs, PAT and relative OSI index ("systemic" and "in penile corpora cavernosa") of the 100 normal cases (control group).

	**d-ROMs values** Carratelli units (Carr. U.) systemic plasma sample	**PAT values** Cornelli units (Cor. U.) systemic plasma sample	**OSI index** systemic plasma sample	**d-ROMs values** Carratelli units (Carr. U.) penile plasma sample	**PAT values** Cornelli units (Cor. U.) penile plasma sample	**OSI index** penile plasma sample
1.	434	3920	**10.1**	356	3465	**9.92**
2.	443	4057	**10**	309	3704	**7.72**
3.	476	4085	**10.7**	348	2939	**11.52**
4.	392	3516	**10.2**	307	2956	**10.02**
5.	385	3843	**9.1**	169	3836	**4.12**
6.	315	2951	**9.7**	294	3327	**8.52**
7.	539	4461	**11.1**	309	3320	**9.02**
8.	373	3651	**9.3**	387	3412	**8.12**
9.	269	2763	**8.8**	299	3217	**8.92**
10.	295	3419	**8.4**	242	3316	**6.92**
11.	329	3544	**8.3**	258	3017	**8.22**
12.	334	3034	**10.1**	219	3302	**6.32**
13.	289	2842	**9.2**	258	3064	**8.12**
14.	323	2756	**10.8**	288	2998	**9.32**
15.	423	3632	**10.7**	315	2895	**10.52**
16.	425	3544	**11**	350	2971	**11.42**
17.	415	3622	**10.5**	261	2891	**8.92**
18.	334	4006	**7.4**	222	2955	**7.22**
19.	299	2984	**9.1**	262	3103	**8.12**

20.	403	3719	**9.4**	288	2821	**9.92**
21.	377	3848	**8.8**	250	2860	**8.42**
22.	441	4613	**8.6**	279	3005	**8.92**
23.	372	3914	**8.6**	312	3416	**8.82**
24.	322	3735	**7.7**	305	4101	**7.12**
25.	275	3283	**7.4**	238	3572	**8.92**
26.	344	3739	**8.3**	313	2981	**10.12**
27.	272	4315	**5.4**	300	3704	**7.72**
28.	382	4461	**7.6**	411	4057	**9.82**
29.	367	4901	**6.5**	437	4324	**9.82**
30.	341	4461	**6.7**	386	5426	**6.82**
31.	401	3824	**9.5**	415	3946	**10.22**
32.	409	4652	**7.8**	325	3112	**10.12**
33.	410	3753	**10**	377	3352	**10.92**
34.	499	4489	**10.2**	451	3854	**11.42**
35.	371	3224	**10.5**	321	3252	**9.52**
36.	513	4922	**9.5**	361	3955	**8.82**
37.	294	2785	**9.6**	261	2551	**9.92**
38.	448	4370	**9.3**	410	3891	**10.02**
39.	363	3712	**8.8**	341	3096	**10.72**
40.	371	3597	**9.4**	372	3263	**11.12**
41.	358	3515	**9.2**	412	3597	**11.12**
42.	318	3055	**9.5**	352	2992	**11.42**
43.	511	4947	**9.4**	485	4295	**10.92**
44.	436	4126	**9.6**	452	3896	**11.32**
45.	401	4075	**8.9**	428	3877	**10.72**
46.	519	4341	**11**	536	4871	**10.72**
47.	430	4080	**9.6**	417	3597	**11.22**
48.	482	4540	**9.7**	431	3690	**11.32**
49.	288	2550	**10.3**	251	2116	**11.52**
50.	402	4134	**8.8**	286	4387	**6.22**
51.	414	3901	**10.1**	343	3454	**9.61**
52.	423	4038	**10**	296	3693	**7.71**
53.	456	4066	**8.3**	335	2928	**11.11**
54.	372	3497	**10.2**	294	2945	**9.61**
55.	365	3824	**9.1**	156	3825	**3.71**
56.	295	2932	**9.7**	281	3316	**8.11**
57.	519	4442	**9.8**	296	3309	**8.61**
58.	353	3632	**9.3**	374	3401	**10.61**
59.	249	2744	**8.8**	286	3206	**8.61**
60.	275	3400	**7.7**	229	3305	**6.61**
61.	309	3525	**8.3**	245	3006	**7.81**
62.	314	3015	**10.1**	206	3291	**5.91**
63.	269	2823	**9.2**	245	3053	**7.71**
64.	303	2737	**9**	275	2987	**8.91**
65.	403	3613	**8.7**	302	2884	**10.11**
66.	405	3525	**9.1**	337	3045	**10.71**
67.	395	3603	**8.6**	248	2880	**8.31**
68.	314	3987	**7.4**	209	2944	**6.71**
69.	279	2965	**9.1**	249	3092	**7.71**
70.	383	3700	**9.9**	273	2810	**9.41**
71.	362	3834	**8.8**	235	2849	**7.91**
72.	426	4599	**8.6**	264	2994	**8.51**
73.	357	3900	**8.6**	297	3405	**8.41**
74.	307	3721	**7.7**	290	4090	**6.71**
75.	260	3269	**7.4**	223	3561	**5.91**
76.	329	3725	**8.3**	298	2970	**9.71**
77.	257	4301	**5.4**	285	3693	**7.41**
78.	367	4447	**7.6**	396	4046	**9.41**
79.	352	4887	**6.5**	422	4313	**9.41**
80.	326	4447	**6.7**	371	5415	**6.51**
81.	386	3810	**9.5**	400	3935	**9.81**
82.	394	4638	**7.8**	310	3101	**9.61**

83.	395	3739	**10**	362	3410	**10.31**
84.	484	4475	**10.2**	436	4023	**10.51**
85.	356	3210	**7.8**	306	3241	**9.11**
86.	498	4908	**9.5**	346	3944	**8.41**
87.	279	2771	**9.6**	246	2540	**9.31**
88.	433	4356	**9.3**	395	3880	**9.81**
89.	348	3698	**8.8**	326	3085	**10.21**
90.	356	3583	**9.4**	357	3252	**10.61**
91.	367	3501	**9.2**	421	3586	**11.41**
92.	327	3041	**9.5**	361	3246	**10.81**
93.	520	4933	**9.4**	456	4284	**10.31**
94.	445	4112	**9.6**	424	3899	**10.51**
95.	410	4061	**8.9**	437	4020	**10.51**
96.	528	4327	**8.6**	467	4860	**9.31**
97.	439	4066	**9.6**	426	4560	**9.01**
98.	491	4526	**9.7**	440	4389	**9.71**
99.	297	2536	**7.9**	340	3890	**8.41**
100.	411	4120	**8.8**	295	4376	**6.41**

NOTE : d-ROMs = derived from the Reactive Oxygen Metabolites; PAT = Plasma Antioxidant Test; OSI = Oxidative Stress Index.
